# Incidence and survival patterns of clear cell renal cell carcinoma from 2000 to 2017: A SEER Database Analysis

**DOI:** 10.7150/jca.105713

**Published:** 2025-02-03

**Authors:** Sijue Zou, Liwen Cui, Pearl Pai, Yiping Lu, XiangYang Li, Gang Wang, Wen Huang, Dan Wang, Nikhat Shaikh, Zhangzhe Peng, Zhuoming Peng, Haiyan He, Zhouning Liao

**Affiliations:** 1 Department of Nephrology, The University of Hong Kong-Shenzhen Hospital, Shenzhen, China.; 2 Department of Nephrology, Xiangya Hospital, Central South University, Changsha, China.; 3 Department of General Surgery, Xiangya Hospital, Central South University, Changsha, China.; 4 III. Department of Medicine, University Medical Center Hamburg-Eppendorf, Hamburg, Germany.; 5 Hamburg Center for Translational Immunology, University Medical Center Hamburg-Eppendorf, Hamburg, Germany.; 6 Department of Respiratory and Intensive Care Medicine, Union Shenzhen Hospital, Huazhong University of Science and Technology, Shenzhen 518000, Guangdong Province, China.

**Keywords:** SEER, Clear cell renal cell carcinoma, Incidence, Age mortality

## Abstract

**Background**: Clear cell renal cell carcinoma (ccRCC) incidence and death have considerably changed in recent years. Our study aimed to investigate the incidence, survival, and tumor characteristics of ccRCC in the year of diagnosis.

**Methods**: Our study participants were selected from the SEER database (2000-2017). Age-standardized incidence rates were calculated to compare incidence rates across time. In addition, we used Kaplan-Meier curves to calculate overall survival (OS) and Cox proportional hazards models to explore risk factors associated with mortality outcomes in patients with ccRCC.

**Results**: In the SEER analysis from 2000 to 2017, the increasing trend in age-adjusted incidence of ccRCC has remained relatively stable over the years, increasing from 2.63 per 100,000 in 2000 to 8.79 per 100,000 in 2017. The increase in the incidence of patients at a localized stage plays a decisive role in the overall increase in the incidence of ccRCC.

**Conclusions**: In the general population, patients diagnosed between 2009-2017 had a higher survival rate than those diagnosed between 2000-2008, which is consistent with all stages of the tumor. The incidence of ccRCC increases steadily with the year of diagnosis, while overall survival has significantly improved.

## Background

Renal cell carcinoma (RCC) is the most common tumor of the urinary system, accounting for approximately 80%-90% of all renal malignancies. In recent years, an increasing number of RCC cases have been diagnosed^1^. In 2020, 431,288 new kidney cancer cases were reported worldwide^2^. ccRCC accounts for approximately 80% of all RCC and is the most common histological type of RCC^3^. It also has an extremely poor prognosis, especially in the case of advanced ccRCC, with a 5-year survival rate of less than 10%^4^.

Many epidemiological studies have reported on the morbidity and mortality associated with RCC. However, few have focused on ccRCC^5^. Although ccRCC is an important component of renal cancer, its epidemiological characteristics are different and an epidemiological description of ccRCC is equally important.

Therefore, based on the SEER database, this study aimed to analyze the changes in the incidence, tumor grade, stage at diagnosis, and mortality of ccRCC over the last 20 years to provide a comprehensive understanding of the epidemiology of clear cell carcinoma and to obtain new findings to identify risk factors, early diagnosis, and disease recognition.

Therefore, we aim to use the SEER database to study the changes in the incidence, tumor grade, stage at diagnosis, and mortality of ccRCC over the last 20 years to provide a comprehensive understanding of the epidemiology of clear cell renal cell carcinoma and how the disease presentation and treatment have changed over the years. SEER database being a comprehensive cancer registry of the USA, its data capturing in 2000-2017 were very consistent and robust.

## Methods

### Population

Patients diagnosed with ccRCC between 2000 and 2017 were collected from the SEER database (https://seer.cancer.gov/) according to 3^rd^ Edition of the International Classification of Tumor Diseases (ICD-O-3). The inclusion criteria were as follows: (1) The first diagnosis was ccRCC. (2) The years at diagnosis were between 2000-2017. Exclusion criteria: (1) The patient had a non-pathological diagnosis. (2) No surgery and surgery unknown. After selection, 101,892 qualified patients with ccRCC were ultimately enrolled in the cohort.

### Covariates and outcomes

We collected the following information: demographic information (including age, gender, race, marital status), tumor characteristics (tumor grade and metastatic stage), and whether surgery had been performed. Tumor grade was based on Fuhrman grade and included grade I (highly differentiated), grade II (moderately differentiated), grade III (poorly differentiated), grade IV (differentiated or undifferentiated), and unknown. Tumor staging was classified as local, regional metastasis, distant metastasis, and unknown. Survival was based on whether death occurred before the end of follow-up. The diagnosis years were further divided into two periods: from 2000 to 2008 (first period) and from 2009 to 2017 (second period). Publicly available data from the SEER database were used for this study.

### Statistical analysis

All analyses were performed using SEER*Stat 8.4.1. Baseline data were all categorical variables and expressed as several cases (percentage). Age-adjusted incidence rates were used to correct for age bias, which visualized the incidence of ccRCC from 2000 to 2017. In addition, Kaplan-Meier curves were used to show survival differences according to year of diagnosis (2000-2008 or 2009-2017) and stratified analysis by tumor stage (local, regional, or metastatic). Multivariate and univariate Cox proportional hazards regression models were used to explore the relationship between overall survival and different variables, including age, gender, race, marital status, tumor grade, tumor stage, and surgical treatment. A two-tailed P value of 0.05 was considered statistically significant.

## Results

### Baseline characteristics

In the SEER analysis from 2000 to 2017, we included a total of 101,892 ccRCC patients (Table 1). of whom 37.85% were female, 85.28% were white, and 62.81% were married. Regarding tumor grading, those in grades I and II accounted for 11.24% and 45.13%, respectively, and grades III and IV combined accounted for a total of 27.95%. Most patients (72.35%) were in localized status, and 9.94% had distant metastases. Of these patients, 93.44% had undergone surgical treatment.

In addition, we also refined the racial background and calculated the age-adjusted incidence of ccRCC in different ethnic populations. It was determined that white people had the highest incidence, followed by American Indian or Alaska Native Americans, further followed by black people, whereas Asian or Pacific Islander had the lowest incidence (Figure S1). The diagnosis of ccRCC peaked in patients aged 70-74 years, with a prevalence of 28.52 per 100,000. This was followed by patients aged 65-69 years, with a prevalence of 26.95 per 100,000 (Figure S2).

### Changes in disease incidence over time

**Figure 1:**
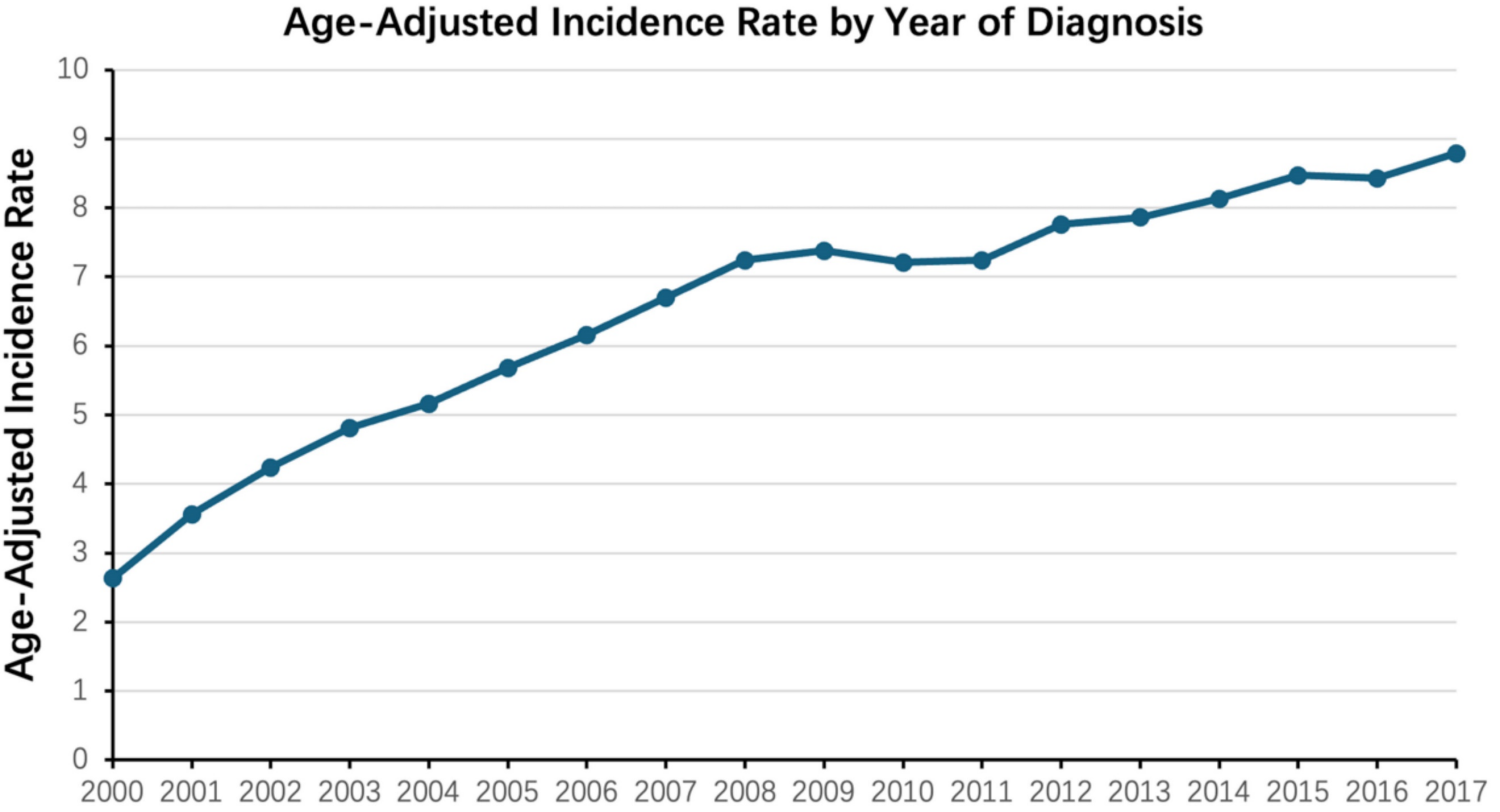
Age-adjusted incidence rate of ccRCC stratified by the year of diagnosis, spanning the period from 2000 to 2017.

From 2000 to 2017, the age-adjusted incidence of ccRCC has increased steadily year by year (Figure 1). The estimated total incidence of ccRCC was 6.58 per 100,000 population, and the annual incidence rate increased from 2.63 per 100,000 in 2000 to 8.79 per 100,000 in 2017.

**Figure 2:**
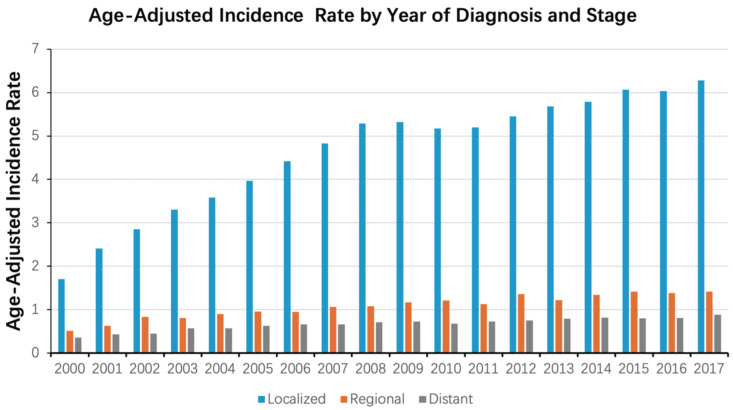
Age-adjusted incidence rate of ccRCC during the period from 2000 to 2017, stratified by both the year of diagnosis and the stage of the disease (localized, regional, distant).

We then analyzed the changes in incidence rate for different tumor stages. The incidence of patients with localized stage tended to increase rapidly over time, while the incidence of patients with regional and distant stage tended to increase slowly overall. Additionally, the proportion of patients in the localized stage far exceeded the other two, regardless of temporal changes (Figure 2). The increase in the incidence of patients with the localized stage plays a decisive role in the overall increase in the incidence of ccRCC.

**Figure 3:**
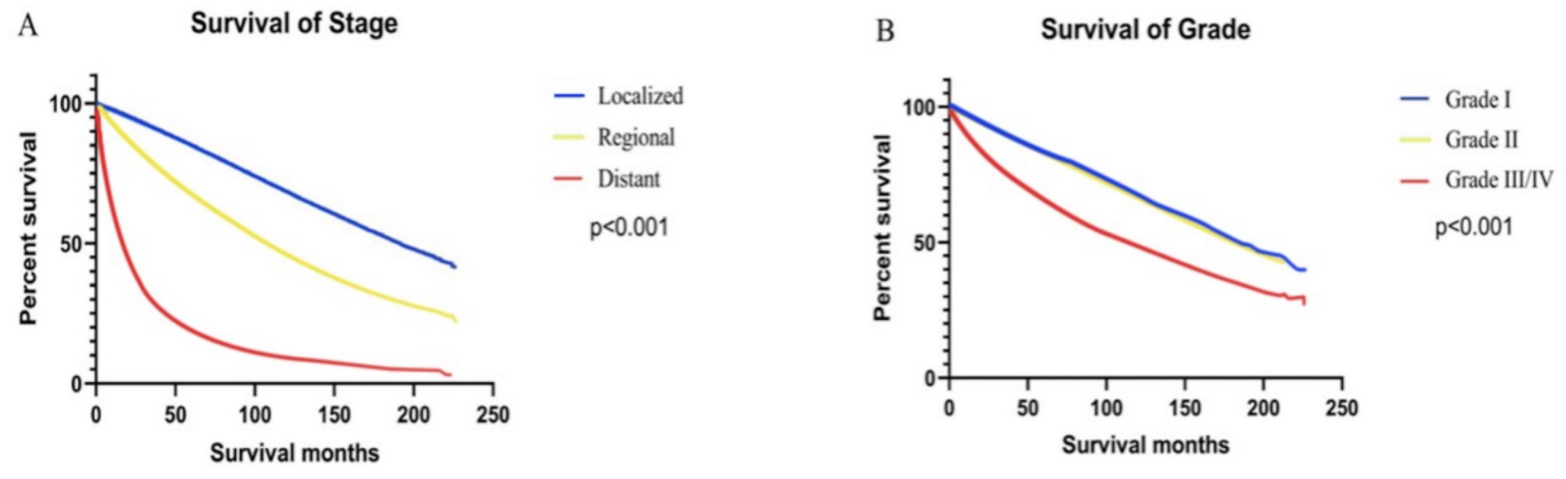
Kaplan-Meier curves of survival rates of patients diagnosed with ccRCC when stratified according to the tumor stage (localized, regional, distant) (A) and tumor grade (Grade I, II, III/IV) (B).

Kaplan-Meier curves showed that patients with different stages or grades of tumors also had different survival rates (P < 0.001). The median overall survival (mOS) for patients with local, regional, and metastatic status was 108, 86, and 16 months, respectively, whereas the 5-year overall survival rates (5-OS rates) were 67.79%, 57.91%, and 18.64%, respectively. Similarly, the mOS for grade I, II, and III/IV ccRCC was 183, 113, and 114 months according to tumor grade. The 5-year OS rate for grade I was 83.31%, grade II 65.85%, and grade III/IV 63.22% (Figure 3).

**Figure 4:**
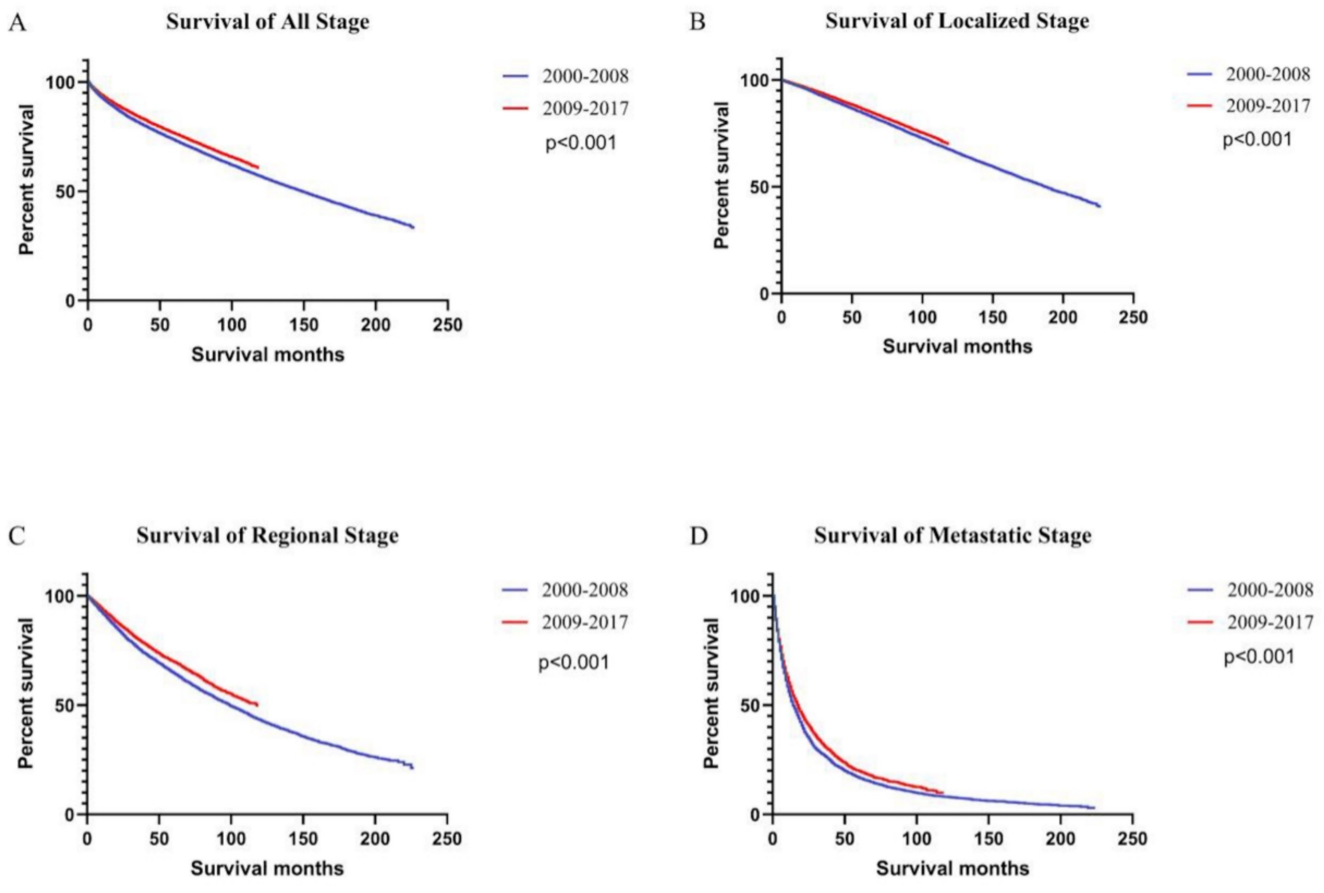
Kaplan-Meier curves of survival rates in patients with ccRCC were generated. Patients were grouped by the year of diagnosis into two periods: 2000 - 2008 and 2009 - 2017. Curves are shown for all stages (A), localized stage (B), regional stage (C), and metastatic stage (D).

Survival rates were higher in 2009-2017 than 2000-2008 period, both for the population as a whole and for patients with all stages of ccRCC (Figure 4). In the general population, survival was consistently higher for patients diagnosed with ccRCC between 2009 and 2017 (5-year OS rate = 76.53%) than those diagnosed between 2000 and 2008 (5-year OS rate = 73.45%) [univariate Cox proportional hazards: hazard ratio (HR): 0.89, 95% confidence interval (CI): 0.88-0.91, P < 0.001]. The 5-year OS rates for patients diagnosed between 2000 and 2008 were 83.83%, 64.74%, and 16.74% for local, regional, and metastatic stages, respectively. The 5-year OS rates for patients diagnosed between 2009 and 2017 were 85.76%, 69.54%, and 19.87%, respectively. Using Univariate Cox proportional hazards regression analysis, the ccRCC patient survival of the second period was better than the first period in localized (HR: 0.91, 95% CI: 0.88-0.93, P < 0.001), regional (HR: 0.86, 95% CI: 0.82-0.90, P < 0.001), and metastatic stages (HR: 0.89, 95% CI: 0.85-0.93, P < 0.001).

Using Multivariable Cox regression analysis, age, gender, race, single status (versus married status), grade III/IV (versus grade I), stage at diagnosis, and surgery (versus no surgery) were all associated with longer OS (Table 2). The Kaplan-Meier curve also showed that the OS of patients treated with surgery was much longer than those who did not receive surgery (Figure S3).

## Discussion

According to population-based research in the USA, the incidence of ccRCC was 3.59 per 100,000 individuals between 1973 and 2014^6^. We have set out to examine the changing ccRCC incidence and trend of cancer mortality in different stages and grades using SEER database of the USA. We have chosen SEER over other cancer registry such as the European Registry because SEER provides more consistent data and reporting, and open access compared to the European registry. We have also chosen a long period for the study between 2000-2017 (divided into two periods) as there had been many changes in diagnostic imaging and therapies which might have affected the cancer epidemiology, and the long period of observation would allow more insight to survival. We believe our study is the first to compare the incidence and mortality of ccRCC of different stages over the last two decades which enable a more comprehensive understanding of the epidemiology of ccRCC.

The incidence of ccRCC, that rose from 2.63 to 8.79 per 100,000 from 2000 to 2017. This rise in the incidence of ccRCC is mainly due to more early localized disease being detected in recent years. Epidemiological studies on ccRCC are of great importance for better understanding of the disease, improving medical practices, and reducing the burden on society.

Our study found an increasing incidence of ccRCC over time, consistent with the overall trend in cancer incidence. The increase in incidence of ccRCC had been noted since the mid-1990s^6^. We have found a steady increase in the incidence of ccRCC between 2000 and 2017 but mainly in the early stage. We have a few hypotheses for such an increase. First, with the social progress and improvement of living standards, the public becomes more aware of their health. There are also more opportunities for health check, and increased availability for medical examination or imaging, such as computed tomography (CT) and magnetic resonance imaging (MRI), which enable early diagnosis^7^-^12^. Second, some lifestyle-related risk factors, such as smoking, alcohol consumption, and obesity are increasing in general, may play a significant role in the development of ccRCC^13^, ^14^. However, this information is not available in the SEER database.

Interestingly, even though our study found an increasing incidence of ccRCC over the years, the mortality rate has decreased. It is likely the rising incidence of grade I tumors has contributed to better survival rates of ccRCC patients. However, the improved survival rates have been observed in ccRCC across all stages. Hence, it is likely the development of more advanced therapy is another key to the improved survival rate of ccRCC patients.

Over the past decade, the treatment strategies for ccRCC have evolved from primarily surgery to the comprehensive use of targeted therapy, immunotherapy, and personalized treatments. While surgery has prolonged the survival of ccRCC patients, metastatic recurrence has been found in up to 30%-40% of postsurgical patients^15^. This was also observed with the patients in our study, in which most patients underwent surgery; however, a proportion of patients still developed metastases. Currently, immune-targeted therapy is an indispensable component of metastatic ccRCC treatment. It mainly includes inhibitors of mammalian targets of rapamycin (mTOR) and vascular endothelial growth factor receptors (VEGFR), such as sunitinib, tivozanib, and temsirolimus^16^. There are also therapies for low to moderate and advanced ccRCC^17^-^21^. The immune checkpoint inhibitors, including nivolumab and atezolizumab, has significantly improved the outcome of patients with advanced ccRCC, particularly those cases that have not been effectively controlled with conventional targeted therapies^22^. By applying targeted therapy and immunotherapy, particularly in advanced and metastatic renal cancer, has improved both the survival rates and quality of life for patients^18^, ^23^.

Many studies have confirmed that tumor stage is an important prognostic factor for ccRCC^24^, ^25^. Neuzillet *et al.* found that genes associated with TNM stage were significant predictors of clear cell carcinoma, even after adjusting for multiple confounding factors^24^. Similarly, we found that with an increase in tumor stage and grade, the prognosis worsened. Therefore, an early identification of tumors and control of risk factors are crucial for tumor prognosis and reduction in disease burden.

In addition to the tumor stage and grade, several other factors are strongly associated with ccRCC prognosis. Age, sex, race, and marital status have been found to influence ccRCC progression and prognosis, with similar results obtained in our study^26^. It has been reported that among patients with clear cell carcinoma, women have a better prognosis than men, which is consistent with our findings^6^, ^27^.

This study has some limitations. Firstly, this was a retrospective study using the SEER database, which only represent the general US population and cannot be extrapolated to the rest of the world. Secondly, in terms of the treatment profile, although the most important treatment approach (surgery) was analyzed, the influence of some factors, such as immunotherapy and treatment of other complications, was not considered. Larger databases and additional studies are required to confirm this hypothesis. Thirdly, the SEER database does not provide details of patients' lifestyle factors, such as smoking and alcohol consumption which may be confounding factors Despite these limitations, the SEER database can offer the most current and comprehensive data for analyzing the trends in incidence and survival of ccRCC over time.

## Conclusion

The incidence of ccRCC has been steadily increasing over time, and most cases are at a localized stage at the time of diagnosis. In addition, patients diagnosed with ccRCC between 2009 and 2017 had higher survival rates than patients diagnosed between 2000 and 2008, across all tumor stages and grades.

## Supplementary Material

Supplementary figures and tables.

## Figures and Tables

**Table 1:** Baseline characteristics of the included patients.

Characteristic	Year of diagnosis		Total (N = 101892)
	2000 - 2008 (N = 35952)	2009 - 2017 (N = 65940)	
Sex			
Female	13906(38.68%)	24661(37.40%)	38567(37.85%)
Male	22046(61.32%)	41279(62.60%)	63325(62.15%)
Race			
White	31054(86.38%)	55836(84.68%)	86890(85.28%)
Black	2476(6.88%)	4838(7.34%)	7314(7.18%)
Other	2287(6.36%)	4804(7.28%)	7091(6.96%)
Unknown	135(0.38%)	462(0.70%)	597(0.58%)
Marital status			
Married	23385(65.05%)	40612(61.59%)	63997(62.81%)
Single	11351(31.57%)	21800(33.06%)	33151(32.54%)
Unknown	1216(3.38%)	3528(5.35%)	4744(4.65%)
Age			
44 years or younger	3397(9.45%)	6185(9.37%)	9582(9.40%)
45 to 64 years	17425(48.47%)	31615(47.95%)	49040(48.13%)
65 years or older	15130(42.08%)	28140(42.68%)	43270(42.47%)
Grade			
I	4795(13.34%)	6657(10.09%)	11452(11.24%)
II	15746(43.80%)	30233(45.85%)	45979(45.13%)
III/IV	8806(24.49%)	19668(29.83%)	28474(27.95%)
Unknown	6605(18.37%)	9382(14.23%)	15987(15.68%)
Stage			
Localized	25863(71.74%)	47851(72.57%)	73714(72.35%)
Regional	6084(16.92%)	11140(16.89%)	17224(16.90%)
Distant	3707(10.31%)	6423(9.74%)	10130(9.94%)
Unknown	298(0.83%)	526(0.80%)	824(0.81%)
Surgery			
No	1889(5.25%)	4720(7.16%)	6609(6.49%)
Yes	34041(94.68%)	61169(92.76%)	95210(93.44%)
Unknown	22(0.07%)	51(0.08%)	73(0.07%)

**Table 2:** Multivariable cox proportional hazards regression for cases diagnosed between 2000 and 2017.

Characteristic	HR	95%CI	P
Sex			<0.001
Female	Reference	Reference	Reference
Male	1.162	1.136-1.190	<0.001
Race			<0.001
White	Reference	Reference	Reference
Black	1.156	1.110-1.204	<0.001
Other	0.902	0.862-0.943	<0.001
Unknown	0.235	0.168-0.329	<0.001
Marital status			<0.001
Married	Reference	Reference	Reference
Single	1.334	1.304-1.366	<0.001
Unknown	0.964	0.911-1.021	0.216
Age			<0.001
44 years or younger	Reference	Reference	Reference
45 to 64 years	2.014	1.897-2.137	<0.001
65 years or older	3.983	3.755-4.225	<0.001
Grade			<0.001
I	Reference	Reference	Reference
II	0.997	0.958-1.037	0.878
III/IV	1.452	1.393-1.512	<0.001
Unknown	1.142	1.094-1.193	<0.001
Stage			<0.001
Localized	Reference	Reference	Reference
Regional	1.849	1.798-1.901	<0.001
Distant	6.206	6.014-6.405	<0.001
Unknown	1.397	1.262-1.547	<0.001
Surgery			<0.001
No	Reference	Reference	Reference
Yes	0.321	0.309-0.334	<0.001
Unknown	0.872	0.648-1.173	0.366

## Abbreviations

RCC: Renal cell carcinoma
ccRCC: Clear cell renal cell carcinoma
OS: overall survival
SEER: Surveillance, Epidemiology, and End Results
HR: hazard ratio
CI: confidence interval
mOS: median overall survival
mTOR: Mammalian targets of rapamycin
VEGFR: vascular endothelial growth factor receptors
