# Supplementary material for: Incidence and survival patterns of clear cell renal cell carcinoma from 2000 to 2017: A SEER Database Analysis
Source: J Cancer. 2025 Feb 3;16(5):1591–7. doi: 10.7150/jca.105713 (PMC11843228; doi:10.7150/jca.105713)
Supplement: Supplementary file 1 — Supplementary figures and tables. [file jcav16p1591s1.pdf]

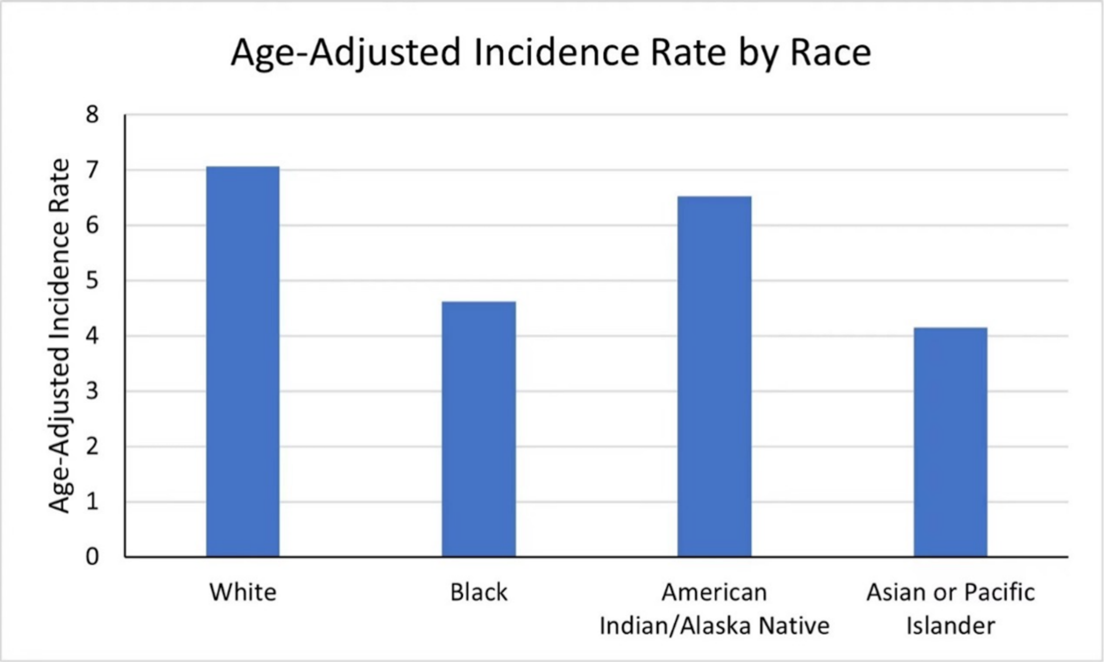

Supplementary figure 1. Age-adjusted incidence rate of ccRCC by race (from 2000 to 2017)

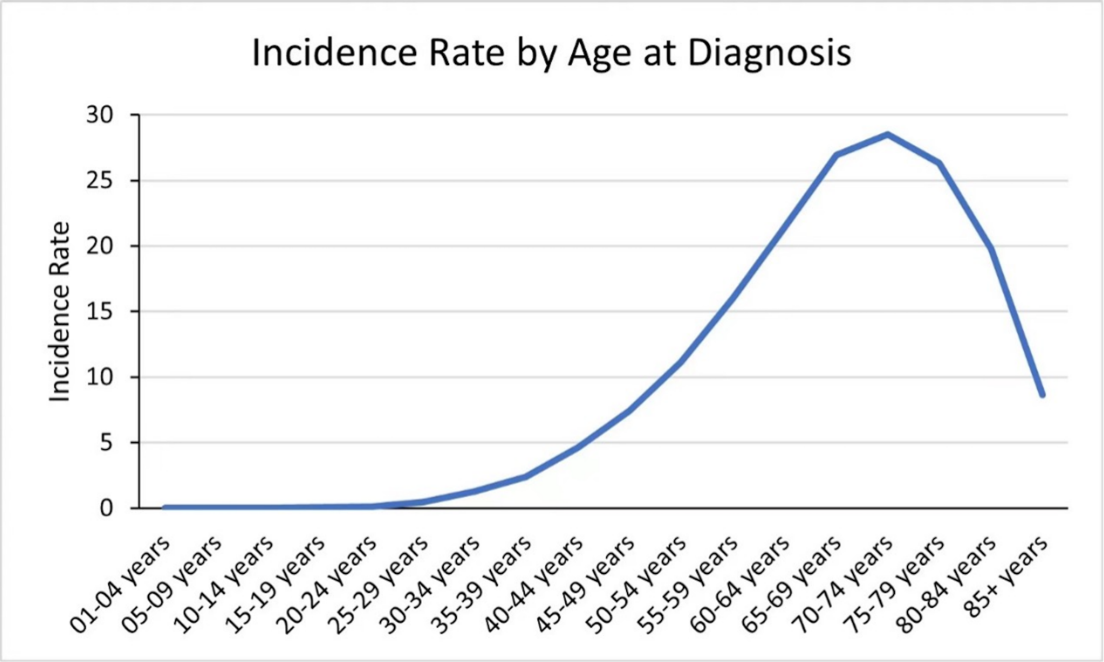

Supplementary figure 2. Age-adjusted incidence rate of ccRCC by age at diagnosis (from 2000 to 2017)

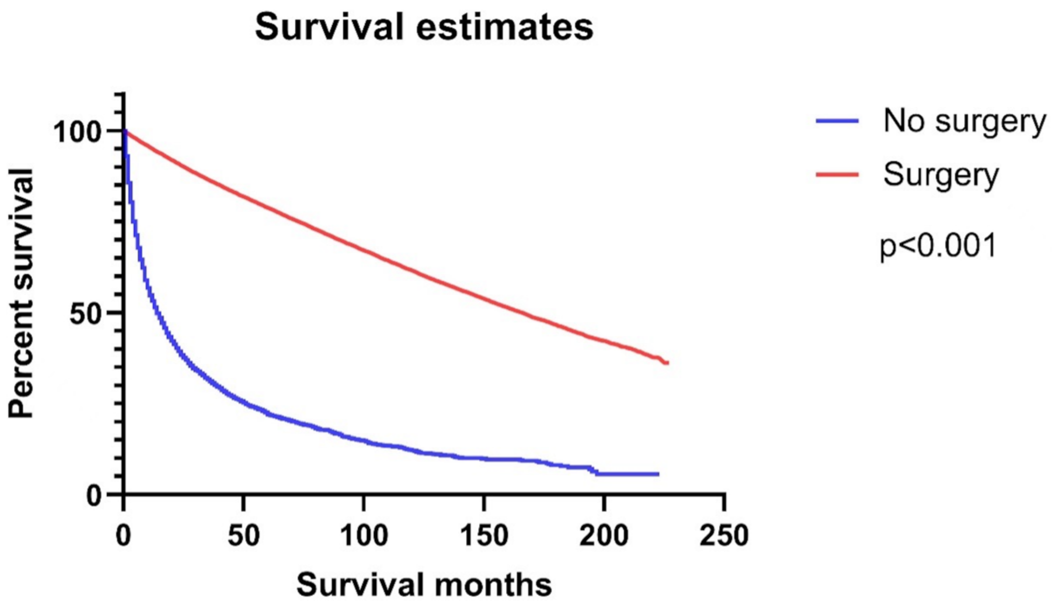

Supplementary figure 3. Kaplan–Meier curves of survival rates in patients with ccRCC according to the surgical treatment
